# A survey of attitudes toward clinical trials and genetic disclosure in autosomal dominant Alzheimer’s disease

**DOI:** 10.1186/s13195-015-0135-0

**Published:** 2015-07-22

**Authors:** Joshua D. Grill, Randall J. Bateman, Virginia Buckles, Angela Oliver, John C. Morris, Colin L. Masters, William E. Klunk, John M. Ringman, for the Dominantly Inherited Alzheimer’s Network

**Affiliations:** Mary S. Easton Center for Alzheimer’s Disease Research, Department of Neurology, University of California, 10911 Weyburn Ave, Ste 200, Los Angeles, CA 90095 USA; Department of Neurology, Washington University School of Medicine, 660 S. Euclid Avenue, Box 8111, St. Louis, MO 63110 USA; Mental Health Research Institute, University of Melbourne, Level 5, Kenneth Myer Building, 30 Royal Parade, Parkville, Victoria 3010 Australia; Department of Neurology, University of Pittsburgh, Room 1422 WPIC, 3811 O’Hara Street, Pittsburgh, PA 15213 USA; Present address: Department of Psychiatry and Human Behavior and UCI Institute of Memory Impairments and Neurological Disorders, University of California, 3206 Biological Sciences III, Irvine, CA 92697-4545 USA

## Abstract

**Introduction:**

Because of its genetic underpinnings and consistent age of onset within families, autosomal dominant Alzheimer’s disease (ADAD) provides a unique opportunity to conduct clinical trials of investigational agents as preventative or symptom-delaying treatments. The design of such trials may be complicated by low rates of genetic testing and disclosure among persons at risk of inheriting disease-causing mutations.

**Methods:**

To better understand the attitudes toward genetic testing and clinical trials of persons at risk for ADAD, we surveyed participants in the Dominantly Inherited Alzheimer’s Network (DIAN), a multisite longitudinal study of clinical and biomarker outcomes in ADAD that does not require learning genetic status to participate.

**Results:**

Eighty participants completed a brief anonymous survey by mail or on-line; 40 % reported knowing if they carried a gene mutation, 15 % did not know but expressed a desire to learn their genetic status, and 45 % did not know and did not desire to know their genetic status. Among participants who knew or wished to know their genetic status, 86 % were interested in participating in a clinical trial. Seventy-two percent of participants who did not wish to learn their genetic status reported that they would change their mind, if learning that they carried a mutation gave them the opportunity to participate in a clinical trial. Nearly all participants responded that they would be interested if an open-label extension were offered.

**Conclusions:**

These results suggest that the availability of clinical trials to prevent ADAD can affect persons’ desire to undergo genetic testing and that consideration can be given to performing studies in which such testing is required.

## Introduction

Biomarker and clinicopathological studies suggest that the pathophysiological process of Alzheimer’s disease (AD) begins long before the onset of cognitive and functional impairment [1–4]. Intervention early in this process may be necessary to successfully alter the underlying biology and clinical progression of disease [5, 6]. Clinical trials of promising preventative therapies, however, require several-year studies of hundreds to thousands of participants to be adequately powered to detect a drug effect [7–9].

Performance of prevention clinical trials in autosomal dominant Alzheimer’s disease (ADAD) avoids some of the challenges associated with sporadic disease. ADAD is characterized by early age of onset (typically in the fourth, fifth, or sixth decade of life) [10] and is also referred to as familial AD. ADAD is caused by mutations in the presenilin (*PSEN1* and *PSEN2*), and amyloid precursor protein (*APP*) genes [11], for which genetic testing is available. Penetrance is near 100 % and the age of symptom onset is largely consistent within families and mutations [12–14]. Mutation carriers demonstrate biochemical and neuroimaging changes that can precede dementia by 15 years or more and may be used to identify windows of time relative to specific pathophysiologic events in disease [4, 15]. ADAD thus presents a unique opportunity to examine experimental preventative therapies for AD: patients can be identified for whom the likelihood of future dementia is essentially certain and therapy can be initiated at predictable times in relation to measurable disease biomarkers. Moreover, in light of the diagnostic reliability and consequent population homogeneity, such studies require fewer patient participants and shorter total study lengths relative to trials in sporadic AD [16, 17].

Despite these strengths, few clinical trials have been performed in ADAD. One challenge to such studies is selecting an appropriate design. An efficient study might enroll only mutation carriers, randomize them to drug or placebo (at a 1:1 ratio), and measure a clinical or biological outcome. Such a study would employ transparent enrollment, including only those who know their mutation status or are willing to learn it in the setting of a trial. Prior studies suggest that less than 10 % of all persons at risk for carrying mutations for ADAD, however, may wish to learn their genetic status [18], creating ethical and logistical challenges to studies enrolling only mutation carriers. Alternatively, trial designs employing blinded enrollment do not require learning genetic status; for example, enrolling all persons at risk for carrying mutations and nonrandomly assigning noncarriers to placebo. These trials may have higher rates of participation but will be larger, more expensive, and carry unique ethical challenges. For example, side effects of investigational treatments might inadvertently lead to unblinding of genetic status for participants that do not wish to know it [17].

The Dominantly Inherited Alzheimer’s Network (DIAN) is a multisite consortium study of ADAD. This longitudinal study enrolls all persons at risk for harboring genetic mutations that result in ADAD. Although the study does not require participants to learn their genetic status, genetic counseling, clinical genetic testing, and disclosure of testing results are offered to all participants at no charge to them. A primary objective of the DIAN is to facilitate clinical trials of promising investigational drugs for ADAD [16]. To assist in designing ADAD clinical trials, a survey was developed to explore the attitudes of DIAN participants toward trials and genetic testing in the setting of these studies. In this paper, we describe the results of this survey and the implications for future clinical trials in ADAD.

## Methods

### Participants

Only participants enrolled in the DIAN were eligible to participate in this survey study. The DIAN is a multisite network that collects clinical, cognitive, imaging, and biochemical assessments from members of pedigrees with known pathogenic mutations in the *PSEN1*, *PSEN2*, or *APP* genes [4, 19]. At the time of survey dissemination, the DIAN included 162 participants. Since then, enrollment has surpassed 400.

### Survey

The data collection instrument was released to DIAN sites as part of a protocol amendment to the larger DIAN study on 28 March 2011. The survey included a brief introductory letter that described the lack of current therapies capable of preventing or slowing AD and the great interest in developing such therapies. The survey was described as providing feedback to investigators to facilitate the design of clinical trials in persons at risk for ADAD. Participants were instructed to complete the survey online (using an included website, username, and password) or via an included hard copy. Survey responses were anonymous; they were not linked to any identifier for the DIAN study. Participants were instructed to use caution to avoid using their name, address, or any other personal identifying information when completing and returning the survey.

**Fig. 1:**
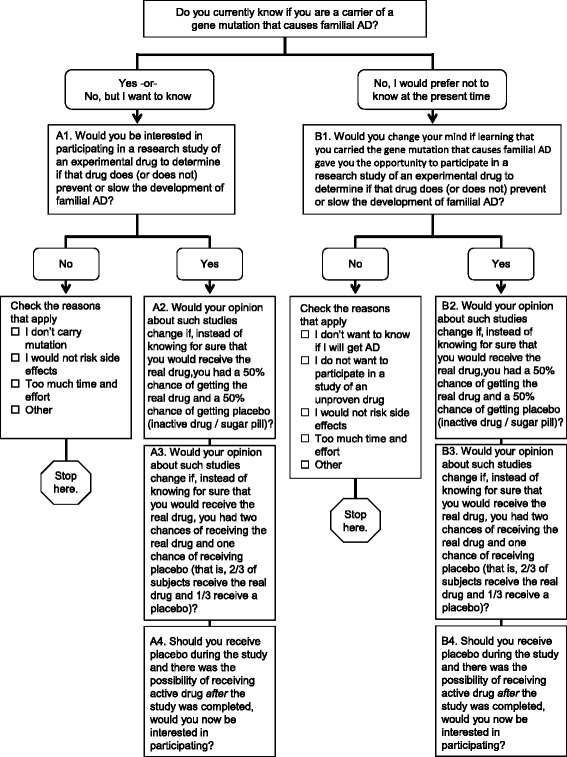
Survey flow diagram illustrating the order of survey questions. Based on their responses, participants were instructed to proceed to specific sections of the survey (e.g., participants that did not wish to know their mutation status were instructed to complete only Section B) or to stop. *AD* Alzheimer’s disease

Figure 1 illustrates the flow of the survey questions, based on participant responses. The first survey item was a multiple-choice question that inquired whether the participant: (1) knew their genetic status, (2) did not know their genetic status but wished to know, or (3) did not know and preferred not to know their genetic status at the present time. Participants who knew or wished to know their genetic status were instructed to answer Question A1, “Would you be interested in participating in a research study of an experimental drug to determine if that drug does (or does not) prevent or slow the development of familial Alzheimer’s disease?” Response choices for this question included “yes” or “no.” If the participant answered “no” they were given the following options and instructed to check all that apply: “I do not carry the mutation that causes the disease in my family,” “I would not want to risk the possibility of side effects,” and “I would not want to spend the extra time and effort.” Additionally, an “other” choice was offered, for which participants could write in responses. Participants answering yes to Question A1 were instructed to answer three additional questions. Question A2 asked “Would your opinion about such studies change if, instead of knowing for sure that you would receive the real drug, you had a 50 % chance of getting the real drug and a 50 % chance of getting a placebo (inactive drug or “sugar pill”)?” (1:1 drug-to-placebo ratio). Question A3 asked “Would your opinion about such studies change if, instead of knowing for sure that you would receive the real drug, you had two chances of receiving the real drug and one chance of receiving placebo (that is, 2/3 of subjects receive the real drug and 1/3 receive a placebo)” (hereafter a 2:1 drug-to-placebo ratio). For both questions, the response choices were: (1) “I would be just as interested,” (2) “I would be less interested,” and (3) “I would be more interested.” Question A4 asked whether “the possibility of receiving active drug after the study was completed” would impact interest in participation and offered (1) “I would now be interested” and (2) “I would still not be interested” as responses.

Participants not interested in genetic testing were instructed to complete Section B. Question B1 asked “If you have chosen not to know your genetic status, would you change your mind if learning that you carried the gene mutation that causes familial Alzheimer’s disease gave you the opportunity to participate in a research study of an experimental drug attempting to prevent or slow the development of familial Alzheimer’s disease?” Participants selecting “no” were asked to select all appropriate reasons that applied: “I do not want to know if I will develop Alzheimer’s disease,” “I do not want to participate in a study of an unproven drug,” “I do not want to risk potential side effects of an unproven drug,” and “I do not want to spend the extra time and effort.” An “other” choice was offered with room for writing in responses. Questions B2, B3, and B4 and their offered response choices were identical to those for Questions A2, A3, and A4.

### Statistical analyses

We examined the frequencies of responses within groups based on the participants’ answer to the initial genetic status question. Statistical comparisons were performed after collapsing those who knew or wished to know their mutation carrier status into a single group. This group was compared with those who preferred not to know their mutation carrier status. Frequencies of participant responses were compared between groups using chi-square (*χ*^2^) tests. Inappropriate responses (e.g., participants who responded that they knew their mutation status but provided responses in Section B) were not included in the statistical comparisons. In the event of missing data, denominators were adjusted to compare proportions of individuals responding to survey items.

### Ethics

The DIAN longitudinal study is approved at each participating site by the local regulatory entity (i.e., institutional review board (IRB) or ethics committee; see Acknowledgements). All participants sign an approved informed consent prior to engaging in any research activities. As part of the DIAN protocol, the current survey was disseminated across sites. For the purposes of the current study, the University of California, Los Angeles, IRB deemed the analysis of the anonymous survey results as not meeting the definition of human subject research.

## Results

### Survey responses

Eighty participants completed the survey; 35 (44 %) completed a paper version and 45 (56 %) completed the survey online. Among participants, 32 (40 %) responded that they knew whether they carried a gene mutation that caused ADAD, 12 (15 %) did not know but wished to learn their genetic status, and 36 (45 %) did not know and preferred not to know their genetic status at the time they completed the survey. There was no difference between those who completed the paper survey versus those completing it online in the proportions that knew their genetic status (*χ*^2^ = 0.67; degrees of freedom = 1, *p* = 0.71).

Table 1 presents the frequency of responses for those who knew or wished to know their genetic status compared with those who did not wish to know their genetic status. We found no differences between the groups in the frequencies of responses.

**Table 1:** Frequencies of survey responses

	Know/want to know	Do not want to know	*χ* ^2^, df, *p* value
*N*	44 (55 %)	36 (45 %)	
Interested in a clinical trial	38 (86 %)	26 (72 %)	*χ* ^2^ = 2.475, df = 1, *p* = 0.116
50–50 drug-to-placebo randomization			
Just as interested	21 (70 %)	14 (58 %)	*χ* ^2^ = 1.75, df = 2, *p* = 0.416
Less interested	9 (30 %)	9 (37 %)	
No longer interested	0 (0 %)	1 (4 %)	
66–33 drug-to-placebo randomization			
Just as interested	27 (90 %)	15 (62 %)	*χ* ^2^ = 5.921, df = 2, *p* = 0.052
Less interested	2 (7 %)	5 (21 %)	
No longer interested	1 (3 %)	4 (16 %)	
Open-label extension study			
Now be interested	29 (100 %)	23 (96 %)	*χ* ^2^ = 1.232, df = 1, *p* = 0.267
Not be interested	0 (0 %)	1 (4 %)	

Item responses are presented for those who know or wish to know their genetic mutation status versus those who do not wish to know (percentages represent the proportion of responses for the specific question). For “interested in a clinical trial,” the phrased question asked those who did not want to know their genetic status if they would be willing to learn it to participate in a clinical trial

*df* degrees of freedom

### Attitudes of those who knew or wished to know their genetic status

Among participants who knew or wished to know their genetic status, 38 (86 %) were interested in participating in a clinical trial. This included 81 % of participants who knew their genetic status and 100 % of those who did not know but wished to know their genetic status. Six participants knew their genetic status but were not interested in participating in a clinical trial; four stated that they did not carry the disease-causing mutation, one was unwilling to risk side effects, and one was unwilling to spend the extra time and effort.

Among those who knew or wished to know their genetic status and were interested in clinical trials, 30 (79 %) responded to the items addressing the placebo ratio. Seventy percent of those who responded were just as interested in a trial with 1:1 randomization to drug or placebo and 90 % were just as interested in a trial with 2:1 randomization. Four (20 %) participants who knew their genetic status were less interested in a 1:1 placebo-controlled trial, compared with five (50 %) of those who did not know their genetic status but wished to (*χ*^2^ = 2.857, degrees of freedom = 1, *p* = 0.091). Only one participant answered that they would not be interested in a placebo-controlled trial. This participant knew their genetic status and stated they would refuse a trial with a 2:1 drug-to-placebo ratio.

Each participant who answered whether the opportunity to participate in an open-label extension study impacted their level of interest stated that they would be interested (*n* = 29, 100 %).

### Attitudes of those who did not want to know their genetic status

Twenty-six (72 %) participants who preferred not to learn their genetic status at the time of completing the survey reported that they would change their mind if learning that they carried a mutation gave them the opportunity to participate in a clinical trial. The eight participants who would not change their mind acknowledged 11 responses why they would decline. Four did not want to know if they would develop AD. Two did not want to risk side effects of an unproven drug. Five selected “other” and wrote in responses. Of these, two said that they did not want to know their status at that time and one stated they were too young to find out their status; one stated that they had young children and would not want to deal with side effects but that they might be willing in future years; one did not want to risk finding out their genetic status only to be placed on placebo; and one stated they would only want to know their status if they knew more about the successes/failures of the trials to this point.

Two participants who acknowledged interest in learning their genetic status and participating in clinical trials did not answer the subsequent questions regarding placebo. Nine of the 24 (37 %) participants who did not want to know their genetic status but would be willing to learn it in the setting of a clinical trial stated that they would be less interested in a trial that had a 1:1 drug-to-placebo randomization and one (4 %) stated that they would no longer be interested. Five (21 %) participants responded that they would be less interested in a trial with 2:1 drug-to-placebo randomization and four (16 %) stated they would no longer be interested. Twenty-three (95 %) participants responded that they “now would be interested” if there was the possibility of receiving active drug after the study was completed.

## Discussion

These results add to a literature describing the willingness of persons at risk for ADAD to participate in clinical research toward developing preventive therapies [17, 20]. Because of the survey’s anonymity and multisite distribution, an exact response rate cannot be calculated. A minimum response rate can be estimated, however, given that at the time the last survey was received there were 353 DIAN participants. Although the generalizability of these results may be limited, some of the findings are noteworthy. Among respondents, 80 % reported that they would be willing to consider participating in a clinical trial, even if it enrolled only persons harboring mutations causing ADAD. Among those who knew or wished to know their genetic status, 86 % endorsed interest in a trial with unspecified design, but the proportion interested dropped when there was a possibility of a placebo. The higher the chance of a placebo, the lower the interest. Interest increased back to the level of the unspecified trial design if open-label extension was included as part of the study. The findings suggest that, in contrast to trials in sporadic AD [21], recruitment may be less of a barrier to trial success in ADAD and support the launch of the DIAN Trials Unit (DIAN-TU) [22].

The DIAN-TU was established to develop, initiate, and coordinate clinical trials in the DIAN cohort [16]. The first such trial, an adaptive-design phase II study of two anti-amyloid-beta therapies with biomarker outcomes that can transition into phase III efficacy measures, is enrolling asymptomatic and very mildly symptomatic persons at risk for ADAD [23]. The trial uses blinded enrollment in which asymptomatic at-risk participants are eligible to participate without the requirement of genetic testing disclosure; noncarriers are nonrandomly assigned to placebo. Mutation carriers are randomized to active drug or placebo at a 3:1 ratio. Although it is theoretically preferable to not mandate that persons undergo genetic testing in order to participate in a prevention study, there are challenges associated with this approach. One potential issue is that mutation carriers who do not know (and may not wish to know) their genetic status might develop adverse effects from the active drug. They would then be made aware of their genetic status, with the potential for psychological reactions. Another issue is the additional costs due to participation of noncarriers, whose data do not contribute to determining whether an intervention is effective. It is therefore important that consideration be given to performing a study enrolling only mutation carriers aware of their status.

In our survey, the frequency with which DIAN participants reported knowing (40 %) or desiring to learn their genetic status (15 %) exceeds previous reports of clinical and research testing for ADAD (often cited as less than 10 %) [17, 18] or other autosomal dominant neurodegenerative diseases (typically less than 25 %) [18, 24–26]. Several factors may contribute to this observation. The DIAN offers free confidential genetic testing, which may reduce barriers related to cost or concerns around stigma [27]. Since the DIAN is a research study, sample bias associated with individuals who are willing to participate in longitudinal clinical and biomarker assessments also may account for differences from previous reports of testing rates. Additionally, the initial papers reporting rates of genetic testing in ADAD are now more than a decade old [18]. The community of ADAD families has therefore had time to learn about and discuss genetic testing [18, 28] and an era of commercial direct-to-consumer genetic testing has brought increased public awareness and understanding of this technology, potentially changing consumer or participant views [29, 30]. Further, since the DIAN was in part initiated with the goal of conducting trials [16], enrollment may be skewed toward individuals interested in therapeutic research and those more likely to wish to learn their genetic status in that setting. Finally, expectation for and the launch of the DIAN-TU prevention trial in 2012 may have changed family member views on genetic testing.

In fact, the majority (72 %) of participants who indicated that they were not interested in learning their genetic status responded that they were willing to do so if it would allow them to participate in a clinical trial of an experimental agent. Together with similar previous results, [18, 20, 31], this finding suggests that trials enrolling only persons who know or are willing to learn their genetic status may be feasible. Such trials would require smaller samples sizes than those of studies enrolling all at-risk persons and would have substantially reduced cost.

The decision whether to undergo predictive genetic testing for ADAD is complex and highly individualized [17, 25, 30]. Asymptomatic patients who choose to learn their mutation status for autosomal dominant neurologic disease may do so to reduce uncertainty and anxiety and to inform life decisions [26, 32]. Although it will require further study, several hypotheses emerge as to why the opportunity to participate in a clinical trial may alter the decision to learn genetic status for some participants. First, it is unclear whether hypothetical responses predict actual behaviors. The proportion of participants in this study who stated they would change their genetic testing decision may or may not be inflated, compared with what would happen in the setting of an actual trial. Next, participants did not undergo systematic and standardized education, counseling, or discussion with researchers prior to completing the survey. The descriptions of trials as testing potentially promising experimental drugs (drugs that aim to slow or stop the progression of AD) may therefore have influenced individuals to state that they would be willing to learn their genetic status despite previously indicating they did not wish to do so. In the setting of fully informed consent (including essential details such as the lack of guaranteed medical benefit and the risk of learning mutation carrier status without such benefit), it is possible that at least some of these individuals would choose not to undergo genetic testing for the sake of enrolling in a trial. Alternatively, the opportunity to take action in the form of research participation may be seen as a benefit of learning genetic status and may alter the risk–benefit profile of the decision, where the risk is learning that one harbors a disease-causing mutation. Taking action, the hope associated with participation and the knowledge that participants are contributing to scientific advancement toward improved medical care for a disease that they and their children may be destined to develop are among the few benefits that investigators can offer individuals at risk for ADAD. For some individuals, these benefits may be enough to warrant learning genetic status when they otherwise would not.

The ideal design for trials in populations at risk for autosomal dominant neurological disorders remains debated. Some investigators conclude that designs requiring disclosure of genetic testing results are coercive [33]. The Belmont report defines coercion as using the threat of harm to ensure compliance [34, 35]. Research participation is voluntary and rarely includes guaranteed health benefits; the decision to decline participation is not harmful. It should also be considered whether designs requiring disclosure result in undue influence; offering excessive or inappropriate reward to obtain compliance [34, 35]. Trials should only test interventions with adequate preclinical and early phase clinical results to support their promise. Access to promising drugs may represent an additional potential benefit for trial participants but is not an inappropriate reward for participation, even if access to therapy is guaranteed [36]. Both ethical and practical issues can thus drive trial design decisions [34]. For example, if inadequate numbers of participants are willing to learn genetic testing results, trials may need to employ blinded enrollment to be feasible. These preliminary results suggest that this may not be the case in ADAD, but future data will be needed to clarify this issue. In all cases, investigators will need to take precautions to ensure informed consent for trials, to minimize risks related to either desired or undesired disclosure of testing results, and to monitor and manage adverse effects of disclosure.

### Limitations

The purpose of this survey was to provide anonymous data to the leadership of the DIAN to assist in trial design and planning. The lack of linked demographic (race, ethnicity, education, geographic residence, etc.) and other data (e.g., family age of onset of disease, participant age relative to family age of onset) prevents analyses for unique patterns or predictors of attitudes toward clinical trials. The survey was not translated to other languages, limiting participation to the English-speaking participants in the DIAN. Since participants were all enrolled in the DIAN observational study, they may not be representative of the greater population of individuals at risk for ADAD, and even within the DIAN we cannot rule out that a particular subset of participants with favorable attitudes toward clinical trials were more likely to complete the survey. It is also possible that those who completed the survey were early enrollers in the DIAN, who may be most motivated to participate in research, including clinical trials. Although these limitations may be minimized since the DIAN participants are likely to represent individuals willing to enroll in ADAD trials, they may still result in an important sample bias.

The survey was brief and instructed participants to stop in the event that particular answers were given to particular questions (see Fig. 1). Data were therefore not collected regarding the willingness to participate in clinical trials among those participants who did not know or wish to know their genetic status and were not willing to learn their genetic status in order to participate in a clinical trial. Ultimately, many of these participants are eligible for and may have enrolled in the DIAN-TU, given that the design does not require learning genetic status. Furthermore, there are several aspects of trial designs that impact willingness to participate, such as risks of adverse events, which were not discussed in the survey. Importantly, adverse events related to the drug under study could unblind mutation status and adverse events related to study procedures are not limited to those receiving therapy. Lastly, it is not clear to what degree these survey responses predict actual behaviors. Of note, however, at least 19 DIAN participants have chosen to undergo genetic testing since the launch of the DIAN-TU.

## Conclusions

The community of ADAD patients and families represents an increasingly informed population that includes many individuals who are motivated to participate in research and can be enrolled in clinical trials to examine drug efficacy at well-defined clinical and pathophysiological stages of disease. Clinical trials will be critical to delaying and preventing the onset of cognitive impairment and dementia in ADAD and may also be instructive toward research and treatment in sporadic AD. This study confirms the strong desire of persons at risk for ADAD to participate in clinical research to develop therapies and suggests that such a desire may affect their decision-making process toward learning genetic status. Some persons who have a stated desire to not learn their genetic status may change their minds in the setting of a clinical trial and essentially all potential participants would be willing to participate if the study offered an open-label extension. The DIAN-TU will provide real-world data on the rate at which persons not previously undergoing testing will change their mind in the context of a clinical trial, and our results suggest that consideration can be given to performing preventive studies enrolling only persons willing to undergo revealing genetic testing prior to enrollment.

## Abbreviations

AD: Alzheimer’s disease
ADAD: Autosomal dominant Alzheimer’s disease
APP: Amyloid precursor protein
DIAN: Dominantly Inherited Alzheimer’s Network
DIAN-TU: Dominantly Inherited Alzheimer’s Network Trials Unit
IRB: Institutional review board
PSEN: Presenilin
